# The Growing Understanding of the Pituitary Implication in the Pathogenesis of Long COVID-19 Syndrome: A Narrative Review

**DOI:** 10.3390/arm92010013

**Published:** 2024-02-14

**Authors:** Ach Taieb, Ben Haj Slama Nassim, Gorchane Asma, Methnani Jabeur, Saad Ghada, Ben Abdelkrim Asma

**Affiliations:** 1 Department of Endocrinology, University Hospital of Farhat Hached Sousse, Sousse 4000, Tunisia; nassimbhs@ymail.com (B.H.S.N.); dargorchene@gmail.com (G.A.); ghada.saad6587@gmail.com (S.G.); asma.benabdelkrim@gmail.com (B.A.A.); 2 Faculty of Medicine of Sousse, University of Sousse, Sousse 4000, Tunisia; jabeur.methnani@gmail.com; 3 Laboratory of Exercise Physiology and Pathophysiology, L.R.19ES09, Sousse 4054, Tunisia

**Keywords:** Post COVID-19 Syndrome, Long COVID-19, pathophysiology, pituitary, corticotrop insufficiency, somatotrop insufficiency

## Abstract

**Highlights:**

**What are the main findings?**

1. COVID-19 may lead to sequelae extending beyond the acute phase of infection, characterized by fatigue and cognitive impairments, forming a novel pathological entity known as Post COVID-19 Syndrome.
2. These sequelae resemble antehypophyseal deficiencies, particularly corticotrop and somatotrop deficiencies, suggesting involvement of the hypothalamo-hypophyseal axis in COVID-19-related long-term symptoms.

**What is the implication of the main finding?**

1. This review underscores the importance of investigating the endocrine system, particularly the pituitary gland, in comprehending and addressing the long-term consequences of COVID-19
2. Recognition of similarities between COVID-19 sequelae and antehypophyseal deficiencies suggests potential implications for targeted diagnostic and therapeutic interventions aimed at addressing hypothalamo-hypophyseal axis abnormalities in affected individuals.

**Abstract:**

Long COVID-19, also known as post-acute sequelae of SARS-CoV-2 infection, is a condition where individuals who have recovered from the acute phase of COVID-19 continue to experience a range of symptoms for weeks or even months afterward. While it was initially thought to primarily affect the respiratory system, it has become clear that Long COVID-19 can involve various organs and systems, including the endocrine system, which includes the pituitary gland. In the context of Long COVID-19, there is a growing understanding of the potential implications for the pituitary gland. The virus can directly affect the pituitary gland, leading to abnormalities in hormone production and regulation. This can result in symptoms such as fatigue, changes in appetite, and mood disturbances. Long COVID-19, the persistent and often debilitating condition following acute COVID-19 infection, may be explained by deficiencies in ACTH and Growth hormone production from the pituitary gland. Corticotropin insufficiency can result in the dysregulation of the body’s stress response and can lead to prolonged feelings of stress, fatigue, and mood disturbances in Long COVID-19 patients. Simultaneously, somatotropin insufficiency can affect growth, muscle function, and energy metabolism, potentially causing symptoms such as muscle weakness, exercise intolerance, and changes in body composition. Recently, some authors have suggested the involvement of the pituitary gland in Post COVID-19 Syndrome. The exact mechanisms of viral action on infected cells remain under discussion, but inflammatory and autoimmune mechanisms are primarily implicated. The aim of our study will be to review the main pituitary complications following COVID-19 infection. Moreover, we will explain the possible involvement of the pituitary gland in the persistence of Post COVID-19 Syndrome.

## 1. Introduction

The most recent pandemic declared to date is the coronavirus disease 2019 (COVID-19). In December 2019, Chinese authorities informed the World Health Organization (WHO) about clusters of viral pneumonias detected in the city of Wuhan [1]. The causative virus is severe acute respiratory syndrome coronavirus 2 (SARS-CoV-2), which is a single-stranded positive-sense RNA virus enveloped in the coronavirus subfamily, highly contagious among humans [1]. COVID-19 defines the disease caused by SARS-CoV-2. As of now, over 769 million people worldwide have been infected with COVID-19, and the WHO has recorded more than 6.9 million deaths [2].

SARS-CoV-2, like other coronaviruses, enters cells via the Angiotensin 2 Conversion Enzyme (ACE2) receptor and Transmembrane Serine Protease 2 (TMPRSS2) [3]. The extensive spectrum of SARS-CoV-2-induced lesions is attributed to the presence of the ACE2 receptor in numerous tissues, including the colon, liver, brain, and various endocrine tissues such as the pancreas, thyroid, and gonads [4,5].

Endocrine disorders associated with COVID-19 have been reported in several studies, exhibiting an endocrine phenotype ranging from clinically paucisymptomatic presentations to potentially life-threatening endocrine emergencies [6,7,8]. The pancreas is the endocrine organ most frequently affected by SARS-CoV-2. COVID-19 is responsible for impairing the glycemic balance in diabetic patients and increasing the incidence and severity of inaugural diabetic ketoacidosis during the pandemic [8,9,10,11].

The thyroid is the second most commonly affected endocrine gland in COVID-19. The most common abnormality in patients infected with SARS-CoV-2 is a decrease in thyroid-stimulating hormone (TSH) and free Tri-iodothyronine. An increased prevalence of thyrotoxicosis and primary hypothyroidism secondary to COVID-19 has also been reported [6,12,13,14,15,16]. Involvement of the adrenal glands by SARS-CoV-2 has been less frequently reported, with preserved adrenal function in the vast majority of patients [17,18,19]. However, primary adrenal involvement by SARS-CoV-2 has been primarily reported due to adrenal hemorrhages and infarctions. It has also been suggested that COVID-19 may play a role in the pathogenesis of Addison’s disease [20,21,22].

Gonadal involvement during COVID-19 has been rarely described. In males, it primarily manifests as orchitis-epididymitis and a tendency towards hypergonadotropic hypogonadism. Disturbances in spermatogenesis have also been reported [23,24,25,26,27]. Regarding ovaries, a few studies have concluded the absence of modification in the ovarian hormonal profile [28,29,30,31].

The central nervous system (CNS) is also a frequent target of SARS-CoV-2. A less explored compartment within the CNS in COVID-19 research, relative to other CNS structures, is the pituitary gland. COVID-19 infection has been associated with hypothalamo–hypophyseal (HH) disorders, such as pituitary apoplexy, diabetes insipidus, and hypophysitis [4,32,33,34,35]. In addition to the ongoing global relevance of the viral infection, the long-term impact of SARS-CoV-2 remains poorly understood. Many patients report the persistence or onset of symptoms, such as fatigue and cognitive impairments, several months after infection. This has led to the definition of a new entity, known as ‘Post COVID-19 Syndrome,’ or more commonly referred to as ‘Long COVID-19’ [36]. The proportion of patients affected by Post COVID-19 Syndrome varies from low percentages to as high as 93% of SARS-CoV-2-infected individuals [37]. Virological and histological hypotheses analyzing these residual symptoms suggest the persistence of certain post-inflammatory lesions, including vascular issues [38]. Other authors have postulated the theory that nano-antioxidants play a role in the pathogenesis of this syndrome [39,40]. However, when closely examining the remaining symptoms in these patients, some are strikingly similar to those seen in antehypophyseal deficiencies, notably corticotrop and somatotrop. Some studies investigating the HH axis have also identified antehypophyseal deficiencies, particularly corticotrop and somatotrop, during the acute phase of COVID-19 infection and in the late post-infectious phase, several months later [4,41].

Recently, some authors have suggested the involvement of the pituitary gland in COVID-19 infection and in Post COVID-19 Syndrome [4]. Certain symptoms could be explained by these pituitary deficiencies. The ACE2 receptor, which enables SARS-CoV-2 entry into cells, is expressed in the HH axis [4]. The exact mechanisms of viral action on infected cells remain under discussion, but inflammatory and autoimmune mechanisms are primarily implicated. Pituitary exploration during infection and follow-up in Post COVID-19 patients has not been systematically established due to the insidious nature of these lesions [4,41].

## 2. COVID-19 and Hypothalamic–Pituitary Diseases

Severe acute respiratory syndrome coronavirus (SARS-CoV) and SARS-CoV-2 belong to the coronavirus family [42]. The principal receptor for SARS-CoV-2, ACE2, manifests a ubiquitous expression across the cellular landscape of endocrine organs, notably prominent in the pancreatic and thyroid tissues [43,44]. This receptor has also been identified in hypothalamo–hypophysial tissues, albeit with a lesser degree of expression compared to other endocrine tissues [45,46].

SARS-CoV has been associated with various endocrinopathies, particularly pituitary-related [45,47]. SARS-CoV-2 has demonstrated a binding affinity to ACE2 10 to 20 times higher than that of SARS-CoV, explaining at least its higher infectious potential [4,48].

Throughout the evolution of the COVID-19 pandemic, several studies have reported hypothalamo–hypophyseal involvement potentially linked to SARS-CoV-2. These mainly include cases of hypophysitis, hypopituitarism, pituitary apoplexies, inappropriate secretion of antidiuretic hormone (SIADH), and diabetes insipidus. Hypothalamic involvement, on the other hand, has been very rarely described [4,8,35,49,50,51,52,53,54]. All these descriptions are summarized in Table 1.

### 2.1. Pituitary Apoplexy

Pituitary apoplexy is a medical emergency that occurs when there is bleeding or impaired blood flow to the pituitary gland, often in the context of a pituitary adenoma. It can cause sudden-onset headaches, visual disturbances, and hormonal imbalances. Patients with severe COVID-19 may be at an increased risk of developing blood clotting disorders, which could potentially lead to conditions like stroke or apoplexy. The virus can trigger an inflammatory response and cause abnormalities in blood coagulation, contributing to the formation of blood clots. The evolution of apoplexy in infected patients is not well evaluated. In common apoplexies, for example, in the study of Falhammar et al., 33 patients had a pituitary apoplexy, 55% of them were men, and the mean age was 46 years. Only 9% of the patients required acute pituitary surgery, while eight patients were operated after more than one week. During follow-up [7.6 ± 4.3 years], none of the hormonal deficiencies regressed, and three patients died from non-related causes [55].

### 2.2. Hypophysitis

Hypophysitis is an inflammation of the pituitary gland and is a rare cause of hypopituitarism. Autoimmune hypophysitis is a known condition in COVID-19, where the body’s immune system attacks the pituitary gland [56]. Determining the actual occurrence rate of hypophysitis following COVID-19 proves to be challenging. Given that a significant number of symptomatic COVID-19 patients undergo glucocorticoid treatment in the early stages of the disease, and in some cases, for extended durations, there exists the potential for a substantial underassessment of hypophysitis diagnoses. In the meta-analysis of Capatina et al., there are only some cases that were reported in the literature, with one case being that of Misgar et al., describing a case of infundibuloneuro hypophysitis, which presented without involvement of the anterior pituitary [4,54].

### 2.3. Syndrome of Inappropriate Antidiuretic Hormone Secretion and Arginine Vasopressin Deficiency

Initial observational studies indicated that around half of COVID-19 patients experienced hyponatremia [57]. However, a retrospective examination of an extensive global registry tracking hospitalized COVID-19 cases, known as the Health Outcome Predictive Evaluation for COVID-19, identified substantially lower frequencies: hyponatremia in 20.5% of cases, hypernatremia in 3.7%. Both conditions were found to be associated with increased mortality and incidences of sepsis [33].

The prevalent cause of hyponatremia, particularly in individuals with COVID-19 pneumonia, was reported to be SIADH. However, whether SIADH directly results from the viral infection remains unclear. It is noteworthy that in various cases, there were reports of newly developed AVP deficiency either during or, more commonly, shortly after COVID-19 infection. This observation raises the prospect of a potential causal association [33].

### 2.4. Central Diabetes Insipidus

Several pathophysiological mechanisms have been proposed to explain CDI secondary to COVID-19. Ong et al. verified the expression of ACE2 in the paraventricular nucleus, making it susceptible to SARS-CoV-2 [58]. Iadecola and colleagues noted the presence of ACE2 and transmembrane protease serine on median eminence capillaries [59]. In a review conducted by Haidar et al. on the involvement of SARS-CoV-2 in central nervous system tissue damage, postmortem examinations have identified the presence of the SARS-CoV-2 genome in the hypothalamus, along with observations of degenerated and edematous neurons [60].

There is a variation in the timeframe between the diagnosis of COVID-19 and the onset of CDI. Yavari A et al. documented a case where central DI manifested six weeks after the initial COVID-19 diagnosis [34].

Similarly, Misgar et al. presented a case in which central DI developed eight weeks after the onset of COVID-19 [54].

### 2.5. Hypothalamic Lesions

There are intricate anatomical and functional interconnection between the hypothalamus and the olfactory bulb. Consistent with prior findings, recent evidence has revealed magnetic resonance imaging (MRI) alterations in the olfactory cortex among COVID-19 patients, underscoring the participation of the olfactory system in viral neuroinvasion [61]. This observation was further elucidated through the utilization of three- and two-dimensional fluid-attenuated inversion recovery images, which delineated cortical hyperintensity in the right gyrus rectus and hyperintensity in the olfactory bulbs [61].

## 3. Hypopituitarism and Post COVID-19 Syndrome

A study conducted by Uhran et al. assessed the anterior pituitary axes of 43 patients, 3 months after the resolution of COVID-19 [41]. Somatotropic and corticotrophic deficits were observed in 46.5% and 16.2% of patients, respectively. Hypogonadism was reported in 9.3% of patients, and hyperprolactinemia in 4.6% of patients [41]. According to the authors, the main hypothesis is that these deficits result from hypophysitis related to COVID-19 [32,50,51,52,53,54,62].

This hypothesis emerged considering the numerous anterior pituitary deficits found during the acute and post-infectious phases of COVID-19, which we will detail below. We will begin by determining the entry and hypothalamo–hypophysial diffusion mechanisms and the pathogenesis of COVID-19-related hypophysitis. Subsequently, we will evaluate the various anterior pituitary axes.

### 3.1. Mechanisms of Entry and Spread Pathway of SARS-CoV-2 in Pituitary Gland

The pathogenesis of hypothalamo–hypophysial lesions related to COVID-19 suggests that the entry of SARS-CoV-2 occurs through the respiratory system. Subsequently, the virus may enter the brain and cause damage to the central nervous system through the following pathways:

- Retrograde neuronal pathway via the nasopharyngeal epithelium and the olfactory nerve.
- Hematogenous pathway, either by crossing the blood–brain barrier or directly through the median eminence at the base of the hypothalamus [63].

Following this, the virus utilizes ACE2 combined with the hypothalamo–hypophysial Transmembrane Serine Protease 2 as a receptor to enter host cells [47,63,64].

The virus has even been isolated in apoplexic pituitary tissue several months after infection, as demonstrated by Albertini et al. This represents the first histological evidence of the presence of SARS-CoV-2 at the pituitary level [62].

### 3.2. Pathophysiology of Hypopituitarism

Several viruses, including Enteroviruses, Herpes Simplex virus, and Varicella-Zoster virus, have been associated with hypophysitis [42].

During the SARS-CoV epidemic, Leow et al. reported corticotropin deficiency in 39.4% of a cohort of 61 surviving patients. The authors concluded that there was a likely secondary hypophysitis due to SARS-CoV that persisted post-infection. Wei et al. found a decrease in immunostaining for corticotrope, somatotrope, and thyrotrope axis among SARS-CoV victims [65,66].

Due to the high homology between SARS-CoV and SARS-CoV-2, the hypothesis of hypophysitis related to COVID-19 is widely supported by various authors. It is thought to result from excessive inflammatory processes or direct viral cytopathic effects in this region [4]. Some authors have raised the possibility that similarly to the thyroid, pancreas, and adrenal glands, autoimmune hypophysitis secondary to COVID-19 may be possible [41]. Supporting the autoimmune origin, Gonen et al. found anti-pituitary and anti-hypothalamic antibodies in 75% of COVID-19 patients with corticotropin deficiencies in their study. Although nonspecific, these antibodies suggest an autoimmune origin of hypophysitis [67]. In a literature review by Krishnappa et al., corticosteroid therapy exceeding 30 mg/day of prednisone equivalent orally for more than 6 weeks was considered the first-line treatment for autoimmune hypophysitis [68]. In severe cases of COVID-19, high doses of glucocorticoids were used for short durations in the majority of hospitalized patients [8,13,65,69].

It is conceivable that insufficient duration of corticosteroid treatment may have allowed temporary remission of hypophysitis before recurrence. This could explain the limited data available on hypophysitis during the pandemic, especially since Krishnappa et al. demonstrated that short-term treatment was associated with poorer long-term outcomes in terms of anterior pituitary deficits [68].

## 4. Corticotropin Deficiency

In a literature review conducted by Vakhshoori et al., the prevalence of adrenal insufficiency among COVID-19 infected patients, ranged from 3.1% to 64.3%. Most studies lacked sufficient data to differentiate between primary and secondary adrenal insufficiencies [70].

The majority of studies focused on cortisol levels during the infection, and only a few actually assessed the corticotropin axis. Subsequently, due to the similarity of certain symptoms between Long COVID-19 and corticotropin insufficiency, and the frequent use of glucocorticoids in hospitalized patients with SARS-CoV-2, the evaluation of the corticotropin axis became more common in the post-infectious phase. This assessment was primarily conducted using the Synacthen Test (ST).

### 4.1. Limitations in the Exploration of Corticotrope Insufficiencies

The majority of studies assessing the corticotropin axis have relied on the ST. The main limitation of these studies, according to the authors, is the absence of the Insulin Tolerance Test (ITT) [41].

The ST has been widely popularized for its milder side effects and the possibility of conducting it in frail subjects for whom the ITT is contraindicated [71].

The consensus of the French Society of Endocrinology emphasizes that the ST can be falsely normal in situations of recent or partial corticotropin deficiencies. In a meta-analysis on this topic conducted by Ospina et al., the authors concluded that the ST (at a dose of 1µg or 250µg) in a suggestive context of corticotropin deficiency does not exclude the diagnosis even if the results are normal [72]. Moreover, there is still uncertainty about the necessary time delay for conducting the ST to assess the corticotropin axis. Many authors have reported falsely reassuring results with the ST for several months in patients with corticotropin deficiencies [71,72,73,74].

Given the previous data, the question arises about the relevance of studies that evaluated the corticotropin axis of COVID-19-recovered patients using the ST after only a few weeks. It is highly likely that partial corticotropin deficiencies may have gone unnoticed in these studies. The main studies evaluating the corticotropin axis during and after COVID-19 are summarized in Table 2.

### 4.2. Pathophysiological Mechanisms of Corticotrope Deficiency in the Post COVID-19 Syndrome

In addition to the hypothesis of secondary hypophysitis due to SARS-CoV-2, other hypotheses have emerged to explain corticotrope impairments in patients recovered from COVID-19.

#### 4.2.1. Corticotrope Impairment Secondary to Corticosteroid Therapy

High-dose corticosteroid therapy was widely used among hospitalized patients during the pandemic. However, it was initiated for short durations (<3 weeks). The ability of synthetic glucocorticoids to induce persistent inhibition of the hypothalamo–hypophysio–adrenal axis even after a brief period of treatment is not clearly predictable due to interindividual pharmacokinetic differences, variations in glucocorticoid sensitivity, and pathophysiological changes in cortisol dynamics in acute and chronic diseases [75,81,82].

Numerous studies have confirmed the lack of a role for corticosteroid therapy in central adrenal insufficiencies, especially the work conducted by Uhran et al., who excluded patients who had received corticosteroid therapy during COVID-19 [4,41,77].

#### 4.2.2. Corticotrope Impairment Secondary to Acth Antibodies

One of the mechanisms proposed by some authors is that, similarly to SARS-CoV, SARS-CoV-2 exhibits molecular mimicry with Adrenocorticotropic hormone (ACTH). It is possible that antibodies produced against SARS-CoV-2 cross-react with the host’s ACTH, contributing to a relative deficiency in ACTH and consequently in cortisol [83,84].

This hypothesis is especially plausible given that viral persistence, which would stimulate the formation of these antibodies, is one of the theories in the pathogenesis of the Post COVID-19 syndrome.

## 5. Somatotropin Deficiency

Generally overlooked during the COVID-19 pandemic, the few studies that have assessed the somatotropic axis during SARS-CoV-2 infection and in the post-infectious phase are summarized in Table 3.

### 5.1. Pathophysiological Mechanisms of Somatotrope Deficiency in the Post COVID-19 Syndrome

#### 5.1.1. SARS-CoV-2 Secondary Hypophysitis

Similarly to the corticotrope axis, secondary somatotropic impairment due to hypophysitis remains the most probable hypothesis for the deficits observed. This is especially true considering that the somatotropic axis is considered the most sensitive to hypothalamo–hypophyseal organic lesions [86].

#### 5.1.2. Giustina Effect

The Giustina Effect refers to a clinical observation that patients with glucocorticoid deficiency (such as in adrenal insufficiency) may also exhibit functional inhibition of Growth Hormone (GH) secretion. The Giustina Effect is reversible with replacement therapy using hydrocortisone, suggesting that correcting the glucocorticoid deficiency can normalize GH secretion in these patients [87]. Although this effect does not account for all the deficits found, it helps explain the predominant and simultaneous impairment of these two axes in the results of Uhran et al. [41].

## 6. Thyrotropic Deficiency

Few studies have reported thyrotropic deficiencies among COVID-19 patients. The impairments are mostly peripheral. In the post-infection phase, thyroid function evaluation was normal in many of the studies [4]. The main studies assessing the thyrotropic axis during and after SARS-CoV-2 infection are summarized in Table 4.

## 7. Gonadotrope Deficiency

The few series reporting gonadotropic impairments during COVID-19 were, according to the authors, the result of a central inhibition of the gonadal axis due to emotional and physical stress during the infection, especially in severe cases. The gonadotropic axis is particularly sensitive to these stimuli [90]. Due to the frequent association of sexual disorders in Long COVID-19, studies have been specifically conducted in patients with these issues, with consistent findings, notably in the work of Al Kuraishy et al. [91].

The main studies evaluating the gonadotropic axis during and after SARS-CoV-2 infection are summarized in Table 5.

### Pathophysiological Mechanisms of Gonadotropic Deficiency in Post COVID-19 Syndrome

Similarly to the other axes, the mechanism for these deficits would most likely be through hypophysitis related to COVID-19 [4]. However, it should be noted that a mechanism of hypothalamic origin was reported by Facondo et al. in a patient with Post COVID-19 syndrome [35].

## 8. Lactotrope Deficiency and Hyperprolactinemia

Hyperprolactinemia is associated with various viral infections, including hepatitis C virus, human immunodeficiency virus, and SARS-CoV [66,95,96].

During the pandemic, different studies assessed the lactotropic axis of patients infected with SARS-CoV-2. The majority reported moderate hyperprolactinemia, and to our knowledge, no study has reported hypoprolactinemia [92].

It is also worth noting that hyperprolactinemia is correlated with a high level of autoantibodies, especially anti-pituitary autoantibodies. It could potentially play a role in the pathogenesis of autoimmune hypophysitis during COVID-19 [97,98].

The main studies evaluating the lactotropic axis during and after COVID-19 infection are summarized in Table 6.

### Pathophysiological Mechanisms of Hyperprolactinemia in the Post COVID-19 Syndrome

The elevation of prolactin during COVID-19 could be explained by various mechanisms, including stress, reduced TSH (thyroid-stimulating hormone), inflammatory state, apoplexies, hypophysitis, and other factors that potentiate prolactin release [98,100].

After the resolution of COVID-19, this moderate elevation of prolactin could be related to the thickening of the pituitary stalk resulting from hypophysitis. In fact, nearly 40% of hypophysitis cases are associated with moderate hyperprolactinemia.

However, prolactin secretion is mediated by multiple factors and is sensitive to numerous stimuli. Therefore, it would be challenging to pinpoint the exact etiology of this hyperprolactinemia.

## 9. Vaccination against SARS-CoV-2 and Hypopituitarism

The question is whether vaccination could play a role in the antehypophyseal deficits found. In a literature review by Ach et al. on this topic, only five cases of hypophysitis and three cases of pituitary apoplexy were reported [101,102].

Its implication in hypopituitarism related to COVID-19 is therefore unlikely [103].

## 10. Conclusions

In conclusion, Long COVID-19, or post-acute sequelae of SARS-CoV-2 infection, extends beyond the acute phase of the illness, impacting various organs and systems, including the crucial endocrine system with a focus on the pituitary gland. This small yet vital gland regulates key hormones essential for numerous bodily functions, encompassing growth, metabolism, stress response, and reproductive processes. The emerging understanding of Long COVID-19 suggests potential implications for the pituitary gland, where the virus’s direct impact can lead to disruptions in hormone production and regulation.

Notably, deficiencies in ACTH and GH production from the pituitary gland may contribute to the persistent and debilitating symptoms observed in Long COVID-19 patients. Following the latest research, corticotrope and somatotrope deficiencies might be implicated in the pathogenesis of the Long COVID-19 syndrome. While acute COVID-19 infection has been associated with transient thyroid abnormalities, there is limited evidence linking Long COVID-19 to persistent thyroid dysfunction. Most cases of thyroid abnormalities during acute infection tend to resolve.

These findings underscore the intricate nature of Long COVID-19, emphasizing the importance of investigating the endocrine system, particularly the pituitary gland, in comprehending and addressing this complex condition. Recognizing and addressing hormone imbalances in Long COVID-19 patients may offer valuable insights into potential therapeutic interventions, providing avenues for improved management and care for those affected by this prolonged and challenging condition.

## Figures and Tables

**Table 1:** Types of lesions occurring to hypothalamo–hypophyseal gland during COVID-19 infection.

Type of Lesions	Authors	Year	Country	Results	Study Description
Pituitary apoplexy	Hazzi et al. [32]	2023	Canada	14 cases	Literature review
Syndrome of Inappropriate ADH secretion	Khidir et al. [33]	2022	Sudan	36% of Hyponatremia	Meta-analysis
Hypophysitis	Capatina et al. [4]	2023	Romania	Not precise	Several cases reported but widely underestimated according to the authors [44,45,46,47,48]
Isolated central diabetes insipidus	Yavari et al. [34]	2022	Iran	1 case	Literature review
Hypothalamitis	Facondo et al. [35]	2022	Italy	5 cases	Literature review

**Table 2:** The main studies assessing the corticotrope axis during the COVID-19 pandemic.

Authors	Year	Country	Patients	Results	Interpretations
Alzahrani et al. [75]	2021	Saudi Arabia	28	Corticotrope deficiency: 28.6%	During the infection No stimulation test
Gu WT et al. [46]	2021	China	114	Reduction in ACTH levels based on the severity of the infection	During the infection No stimulation test
Das et al. [76]	2021	India	84	Reduction in ACTH levels based on the severity of the infection	During the infection No stimulation test
Gonen et al. [67]	2022	Turkey	49	Corticotrope deficiency: 8.2%	During the infection ST
Clark et al. [77]	2021	United Kingdom	70	No corticotrope deficiency	Post-infection ST
Uhran et al. [41]	2022	Turkey	43	Corticotrope deficiency: 16.2 %	Post-infection ST
				9.3%	Glucagon stimulation test
Sunada et al. [78]	2022	Japan	186	Decrease in the ACTH/cortisol ratio	Post-infection Suggests a decrease in the reactivity of the hypothalamo–adrenal axis
Klein et al. [79]	-	United States	215	Low cortisol levels associated with normal ACTH	Post-infection Corticotropic Involvement in the fatigue of Long COVID-19
Ach et al. [80]	2023	Tunisia	64	The proportion of corticotrope-deficient individuals is higher among Long COVID-19 patients (G2) compared to recovered COVID-19 patients (G1) (G1: 6.3% vs. G2: 28.1%)	Post-infection ITTCorticotropic involvement in Long COVID-19

Adrenocorticotropic Hormone (ACTH); Synacthen Test (ST); Insulin Tolerance Test (ITT).

**Table 3:** Main studies assessing the somatotropic axis during the COVID-19 pandemic.

Authors	Year	Country	Patients	Results	Interpretations
Gu WT et al. [46]	2021	China	114	GH levels similar to the control group.	During the infection No stimulation test
Baykan et al. [85]	2022	Turkey	456	Low levels of GH and IGF-1 in severe forms.	During the infection No stimulation test
Sunada et al. [78]	2022	Japan	186	Lower levels of GH in post-infectious patients experiencing fatigue.	Post-infection No stimulation test
Uhran et al. [41]	2022	Turkey	43	Somatotrop deficiency: 46.5%.	Post-infection Glucagon stimulation test
Ach et al. [80]	2023	Tunisia	64	The proportion of somatotrope-deficient individuals is higher among Long COVID-19 patients (G2) compared to recovered COVID-19 patients (G1). (G1: 31.3% vs. G2: 59.4%)	Post-infection ITTSomatotropic involvement in Long COVID-19

GH: Growth Hormone; IGF-1: Insulin-Like Growth Factor; Insulin Tolerance Test (ITT).

**Table 4:** Main studies assessing the thyrotropic axis during the COVID-19 pandemic.

Authors	Year	Country	Patients	Results	Interpretations
Das et al. [76]	2021	India	84	Thyrotropic deficiency: 28.5%	During infection
Chen et al. [88]	2021	China	50	Thyrotropic deficiency: 6%	During infection
Clark et al. [77]	2021	United Kingdom	70	No thyrotropic deficiency	Post-infection
Uhran et al. [41]	2022	Turkey	43	No thyrotropic deficiency	Post-infection
Lui et al. [89]	2023	China	250	No thyrotropic deficiency	Post-infection

**Table 5:** Main studies assessing the Gonadotropic axis during the COVID-19 pandemic.

Authors	Year	Country	Patients	Results	Interpretations
Das et al. [76]	2021	India	84	Gonadotropic deficit: 58.3% in severe forms.	During infection
Cai et al. [92]	2022	China	3369	No significant changes in sex hormones except for an elevation of LH.	During infection Meta-analysis
Moreno-Perez et al. [93]	2021	Spain	143	Gonadotropic deficiency: 22.3%.	Post-infection
Al kuraishy et al. [91]	2022	Iraq	39	Lower levels of LH and testosterone compared to controls.	Post-infection Cohort of 39 Long COVID-19 patients with erectile dysfunction
Kamil et al. [94]	2022	Iraq	50	Persistent gonadotropic deficit in 30% of patients.	Post-infection Follow-up of 50 patients over 1 year.

LH: Luteinizing Hormone.

**Table 6:** Main studies assessing the Lactotrope axis during the COVID-19 pandemic.

Authors	Year	Country	Patients	Results	Interpretations
Das et al. [76]	2021	India	84	Hyperprolactinemia: 42.1%	During infection
Cai et al. [92]	2022	China	3369	No modification in prolactin levels	During infection Meta-analysis
Kumar et al. [99]	2021	India	235	Hyperprolactinemia: 8.5%	During infection
Moreno-Perez et al. [93]	2021	Spain	143	Hyperprolactinemia: 8.3%	Post-infection
